# Supplementary material for: MetaRibo-Seq measures translation in microbiomes
Source: Nat Commun. 2020 Jun 29;11:3268. doi: 10.1038/s41467-020-17081-z (PMC7324362; doi:10.1038/s41467-020-17081-z)
Supplement: Supplementary file 10 — Supplementary Data 7 [file 41467_2020_17081_MOESM10_ESM.zip › File2/Confidence_High_Taxonomy/439802_out.krona.html]

Javascript must be enabled to view this page.

members
magnitude
magnitudeUnassigned
count
unassigned
taxon
rank

439802\_out

50

2
50
superkingdom

phylum
976
50

50
200643
class

50
171549
order

family
2
815

1
816
2

SRS045004\_contig\_number\_13168
genus


SRS058070\_contig\_number\_16786
species
357276
1

1853231
48
family

283168
10
3

SRS023526\_contig\_number\_contig-100\_2632.197095SRS063370\_contig\_number\_contig-100\_1025.58379SRS077294\_contig\_number\_9936
genus

2292236
2

SRS098827\_contig\_number\_7758SRS143876\_contig\_number\_25240
species

3
2292048
species

SRS013800\_contig\_number\_14000SRS075341\_contig\_number\_15209SRS147346\_contig\_number\_51559

2
28118
species

SRS024132\_contig\_number\_23013SRS078419\_contig\_number\_35880

genus
574697
38

21
544645
species

SRS011239\_contig\_number\_24991SRS013098\_contig\_number\_36077SRS015694\_contig\_number\_2539SRS018351\_contig\_number\_contig-100\_16.98463SRS020233\_contig\_number\_38648SRS049959\_contig\_number\_42754SRS053398\_contig\_number\_contig-100\_836.149718SRS055017\_contig\_number\_26618SRS056259\_contig\_number\_3817SRS056273\_contig\_number\_24493SRS058770\_contig\_number\_3900SRS062654\_contig\_number\_4913SRS075078\_contig\_number\_25667SRS077024\_contig\_number\_4589SRS098644\_contig\_number\_45029SRS103987\_contig\_number\_32175SRS1041039\_contig\_number\_9357SRS1041134\_contig\_number\_11903SRS142599\_contig\_number\_12888SRS144537\_contig\_number\_29016SRS147271\_contig\_number\_36847

17
1472416
species

SRS014613\_contig\_number\_5998SRS015431\_contig\_number\_13001SRS016018\_contig\_number\_15845SRS016132\_contig\_number\_1864SRS016335\_contig\_number\_18950SRS018836\_contig\_number\_13672SRS019068\_contig\_number\_11940SRS019267\_contig\_number\_2864SRS020869\_contig\_number\_22054SRS049896\_contig\_number\_29574SRS050925\_contig\_number\_33394SRS064276\_contig\_number\_29267SRS104311\_contig\_number\_contig-100\_363.244762SRS143342\_contig\_number\_12732SRS143780\_contig\_number\_23195SRS145497\_contig\_number\_20092SRS148196\_contig\_number\_37818
